# Increasing the Price of Alcohol as an Obesity Prevention Measure: The Potential Cost-Effectiveness of Introducing a Uniform Volumetric Tax and a Minimum Floor Price on Alcohol in Australia

**DOI:** 10.3390/nu12030603

**Published:** 2020-02-26

**Authors:** Ella Robinson, Phuong Nguyen, Heng Jiang, Michael Livingston, Jaithri Ananthapavan, Anita Lal, Gary Sacks

**Affiliations:** 1 Global Obesity Centre (GLOBE), Institute for Health Transformation, Deakin University, Geelong, VIC 3220, Australia; phuong.nguyen@deakin.edu.au (P.N.); jaithri.ananthapavan@deakin.edu.au (J.A.); anita.lal@deakin.edu.au (A.L.); gary.sacks@deakin.edu.au (G.S.); 2 Deakin Health Economics (DHE), Institute for Health Transformation, Deakin University, Burwood, VIC 3125, Australia; 3 Centre for Alcohol Policy Research (CAPR), School of Psychology and Public Health, La Trobe University, Bundoora, VIC 3086, Australia; jason.jiang@latrobe.edu.au (H.J.); m.livingston@latrobe.edu.au (M.L.); 4 Centre for Health Equity, Melbourne School of Population and Global Health, University of Melbourne, Carlton VIC 3053, Australia

**Keywords:** obesity prevention, alcohol policy, taxation, minimum pricing, economic modeling

## Abstract

The objective of this study was to estimate, from an obesity prevention perspective, the cost-effectiveness of two potential policies that increase the price of alcohol in Australia: a volumetric tax applied to all alcohol (Intervention 1) and a minimum unit floor price (Intervention 2). Estimated changes in alcoholic drink consumption and corresponding changes in energy intake were calculated using the 2011–12 Australian Health Survey data, published price elasticities, and nutrition information. The incremental changes in body mass index (BMI), BMI-related disease outcomes, healthcare costs, and Health Adjusted Life Years (HALYs) were estimated using a validated model. Costs associated with each intervention were estimated for government and industry. Both interventions were estimated to lead to reductions in mean alcohol consumption (Intervention 1: 20.7% (95% Uncertainty Interval (UI): 20.2% to 21.1%); Intervention 2: 9.2% (95% UI: 8.9% to 9.6%)); reductions in mean population body weight (Intervention 1: 0.9 kg (95% UI: 0.84 to 0.96); Intervention 2: 0.45 kg (95% UI: 0.42 to 0.48)); HALYs gained (Intervention 1: 566,648 (95% UI: 497,431 to 647,262); Intervention 2: 317,653 (95% UI: 276,334 to 361,573)); and healthcare cost savings (Intervention 1: $5.8 billion (B) (95% UI: $5.1B to $6.6B); Intervention 2: $3.3B (95% UI: $2.9B to $3.7B)). Intervention costs were estimated as $24M for Intervention 1 and $30M for Intervention 2. Both interventions were dominant, resulting in health gains and cost savings. Increasing the price of alcohol is likely to be cost-effective from an obesity prevention perspective in the Australian context, provided consumers substitute alcoholic beverages with low or no kilojoule alternatives.

## 1. Introduction

The World Health Organization (WHO) recommends fiscal policies to restrict the consumption of unhealthy foods and drinks as part of a suite of measures to address overweight and obesity globally [1]. Over the past several years, a growing number of countries and jurisdictions have introduced fiscal policies to help curb rising obesity rates, primarily focused on taxes on sugar-sweetened beverages (SSBs), which are a risk factor for obesity and obesity-related conditions including diabetes, several cancers, and cardiovascular disease [2]. Whilst alcoholic beverages are typically high in kilojoules [3], the taxation of alcohol has not been a focus of obesity prevention efforts [4]. Nevertheless, the taxation of alcoholic beverages is commonplace globally as a way to generate government revenue and prevent the significant harms associated with alcohol [5]. Alcohol is a risk factor for a number of chronic conditions as well as a major contributor to accidents, injuries, violence, crime, mental and behavioral disorders and alcohol dependence [6,7]. However, in countries like Australia, the current system for the taxation of alcohol does not adequately address the negative externalities associated with alcohol [8] including those potential externalities related to obesity. 

In Australia, alcoholic drinks contribute the most (out of all food and drink categories) to discretionary food or drink intake at the population level, amounting to 4.8% of daily energy intake overall [9]. In comparison, confectionery and cereal bars contribute around 2.8% and soft drinks contribute 1.9% of daily energy intake [9]. There are a number of studies that suggest an association between alcohol intake and increased energy intake [3,10,11]. A recent systematic review and meta-analysis by Kwok et al. (2019), which included 22 studies, found that alcohol consumption significantly increased both food energy intake and overall energy intake, and that intake of food was not reduced to compensate for the energy consumed from alcohol [11]. Another recent systematic review by Cummings et al. (2019) found that across 30 studies, low and moderate alcohol use was linked to greater dietary intake [10]. Considering the significant contribution of kilojoules from alcohol in the diet, and evidence that the consumption of alcohol is likely to lead to increased energy intake, a reduction of alcoholic beverage intake represents a potentially important component of a comprehensive obesity prevention strategy.

There has been extensive research investigating the impact of pricing policies on the consumption of alcohol, which has been widely shown to be highly effective for reducing alcohol consumption [12,13]. In Australia, alcoholic beverages are currently taxed in a complex way whereby different types and tiers of taxation apply to different types of alcohol. The Wine Equalization Tax (WET) is applied to wine, and equates to a 29% tax on the wholesale price of wine [14]. An excise tax of varying amounts is applied to all other alcoholic beverages, dependent on their alcohol content, alcohol type, and packaging [14]. The current system allows certain types of alcohol such as cask wine to be taxed at a relatively low rate, and does not encourage low risk drinking behaviors. 

There have been numerous calls to overhaul the current alcohol taxation system in Australia and there are several pricing approaches available to policy makers looking to reduce harm from alcohol consumption. One option is volumetric taxation, which taxes alcohol based on alcohol content and is applied equally across alcoholic drinks. Previous modeling studies have shown this approach to be cost-effective and leads to significant health gains [15,16]. A noTable 2009 review of Australia’s future tax system, ‘The Henry Review’, recommended replacing the current tax system with an evidence based uniform volumetric tax on all alcoholic beverages (Recommendation 71) as an effective approach for reducing harm from alcohol in Australia [8]. Another option is minimum pricing, which sets a minimum unit price below which a standard drink of alcohol cannot be sold. Minimum pricing is not a tax, however, works to increase the price of very low cost alcohol. International studies have indicated that this is likely to be effective at reducing alcohol consumption, particularly amongst harmful drinkers [17,18]. In October 2018, the Northern Territory (NT) government introduced a minimum floor price of AUD($)1.30 per standard drink on all alcohol, in response to recommendations put forward by the Alcohol Policies and Legislation Review Final Report into alcohol-related harm in the NT [19].

While governments around the world have adopted a range of measures aimed at alcohol reduction, the potential impact of alcohol-reduction interventions as an obesity prevention measure has not previously been quantitatively explored. This study aimed to model the potential cost-effectiveness, from an obesity prevention perspective, of two potential pricing interventions designed to increase the price of alcohol in Australia: a uniform volumetric tax and a minimum unit floor price.

## 2. Methods

### 2.1. Interventions to be Modeled

Pricing interventions were selected based on alcohol pricing policy approaches from the literature, taking into account policy relevance to Australia [8,15,16,17,18,19].

Intervention 1 was the introduction of a uniform volumetric tax, at a rate equivalent to a 10% increase in the baseline tax rate for off-premises spirits (baseline $0.97 per standard drink) (20). This was equal to $1.07 per standard drink across all alcoholic drink categories. A 10% increase was chosen due to its broad plausibility and likelihood to have an impact on health outcomes. Baseline tax rates were specified as the 2013 rates for each alcoholic drink category, due to relevant price estimates being based on 2013 data [20]. This intervention was assumed to be implemented at the federal level in Australia, and applied uniformly in each jurisdiction. This intervention focused on correcting for negative externalities associated with alcohol, and as such, the pass-through rate of the tax was set at 100% (i.e., full cost borne by the consumer).

Intervention 2 was a minimum unit floor price applied to all types of alcoholic drinks at a rate of $1.30 per standard drink. This rate was chosen for its policy relevance following the recent introduction of a $1.30 floor price in the Northern Territory. This is likely to reflect any future policy interventions around minimum pricing in Australia. This intervention would affect only those alcoholic drinks that are currently sold under $1.30 per standard drink. This intervention was assumed to be legislated and implemented on a mandatory basis in each state and territory concurrently. 

Both interventions were modeled across the 2010 Australian population aged 15 and older. 

### 2.2. Effect of Interventions on Alcohol Consumption and Body Weight

Baseline alcohol prices were taken from the 2013 Australian estimates [21] for each type of alcohol and purchase location, specified as on-premises (purchased in licensed premises such as bars, clubs, restaurants, and hotels) or off-premises (alcohol bought from liquor stores and retail outlets). Baseline tax rates were specified as the 2013 rates for each alcoholic drink type, due to relevant price estimates being based on 2013 data [20]. 

Baseline mean daily intake of different types of alcoholic drinks for 5-year age and sex groups were extracted from the 2011–12 Australian Health Survey (AHS) data [9]. As cask wine was not specified in this dataset, intake was calculated based on the proportion of wine sold as a soft pack using 2011–2012 Australian Bureau of Statistics data [22]. Consumption was further divided into on-premises and off-premises consumption, based on available macro-level purchasing data from Euromonitor International (Australia Off trade vs. On-trade data 2012–2017) [23]. 

Recently published Australian own- and cross-price elasticity estimates for off-premises and on-premises alcoholic drinks [21] were used to calculate the relative change in alcohol purchase and consumption post-intervention. It was assumed that these price data and elasticity estimates would apply to the 2010 population. Where price elasticities from alcohol drinks in the AHS were not available, we applied the price elasticity of other equivalent alcoholic drinks (e.g., price elasticity of bottled wine was applied to all wine categories; the price elasticity of spirits was applied to liqueurs, cocktails, mixed drinks; the price elasticity of regular beer was applied to cider). In the base case analyses, we assumed no substitution to non-alcoholic beverages or foods, based on the lack of clear evidence of any such substitution. This was varied in a scenario analysis in which we modeled the effectiveness of both interventions after alcoholic beverages were substituted with SSBs. For this analysis, SSBs were defined as full sugar cola, the mean kilojoule content of which was determined through the Australian Food, Supplement, and Nutrient Database (AUSNUT) 2011–13 food nutrient database [24]. It was assumed that a standard serving of alcohol (e.g., can of beer, glass of wine) would be substituted with a standard serving of full sugar SSB. Standard servings of alcohol were estimated using the Australian Government standard drinks guide [25], and a standard serving of full sugar SSB (equivalent to a 375 mL standard can) was based on data from the AUSNUT 2011–13 nutrient database. 

AUSNUT 2011–13 food nutrient data [24] was also used to determine the mean kilojoule content of each alcoholic drink type and this was used to calculate the baseline daily energy contribution from alcohol in the diet. Change in alcoholic drink consumption post-intervention was calculated for each alcoholic drink type, and for each age and sex group. The change in daily energy intake (in kilojoules) were converted to a corresponding change in mean body weight using a validated energy balance model that quantifies the changes in energy expenditure to changes in body weight [26]. Corresponding changes to body mass index (BMI) were calculated using the AHS population height data. It was assumed that changes in BMI were maintained over the life time of the population.

### 2.3. Health Outcomes Modeling

A previously developed model, the ACE-Obesity Policy model, was used to estimate the health outcomes that resulted from changes to BMI [27]. 

The ACE-Obesity Policy model is a multi-state, multiple cohort life table model that simulates the incidence, prevalence, and mortality related to nine obesity related diseases (i.e., type 2 diabetes, hypertensive heart disease, ischemic heart disease, stroke, osteoarthritis of the hip and knee, kidney cancer, colorectal cancer, endometrial cancer, and breast cancer) over the lifetime of the 2010 Australian population. The ACE-Obesity Policy model has been used to evaluate the economic credentials of various obesity policies in Australia [28,29,30,31] and details of the model have been previously published [27,32,33]. In brief, the model uses the prevalence of overweight and obesity taken from the Australian Health Survey 2010 [34] and relative risk estimates from the Global Burden of Disease (GBD) 2010 [35] to calculate the population impact fractions for nine obesity-related diseases. Disability weights from the GBD study [35] were used to calculate the morbidity associated with the included diseases. The change in the epidemiology of these diseases was used to calculate the total morbidity and mortality changes (quantified as Health Adjusted Life Years (HALYs)) of interventions that change the BMI profile of the Australian population. The model calculates the HALYs using disability weights from the global burden of disease study to weight disease states in adults and utility weights to value health states associated with BMI status in children. The model also calculates the healthcare cost savings (cost offsets) resulting from the intervention compared to a no intervention comparator (where the distribution of BMI for population remained unchanged) was estimated. 

Health outcomes associated with changes in alcohol consumption independent of changes in BMI were not included in the analyses.

### 2.4. Costing

#### 2.4.1. Costs to Government

Australian estimates of parliamentary legislation costs were based on previous estimates of sugar-sweetened beverage (SSB) tax legislation costs [32]. Implementation, compliance, and monitoring costs were based on costings from the 2011–12 New South Wales (NSW) Fast Food Labeling review report, which outlined the government costs associated with introduction of a menu board labeling scheme in NSW [36]. In line with the NSW Government approach, we costed monitoring for 7% of the total number of liquor retailers across Australia, for the duration of the intervention. Additionally, we included the cost of running a nation-wide education campaign to provide information and resources to consumers and industry regarding the intervention, also based on the estimated costing for the consumer fast food labeling campaign run by the NSW Government [36]. In relation to the implementation of the minimum floor price intervention, additional costs included the provision of government advice to major liquor retail chains in Australia in the first year of implementation. 

Government revenue from the introduction of a uniform volumetric tax intervention was calculated, but reported separately from the cost-effectiveness analyses.

Data on healthcare costs were obtained from the Australian Institute of Health and Welfare (AIHW) for 2001 [37]. Cost per prevalent or incident case and all other intervention costs were adjusted to 2010 prices using either the total health price index or gross domestic product index [38]. 

#### 2.4.2. Costs to Industry

Due to the limited data around the real-world costs of either intervention, compliance costs for industry were based on estimates from the Scottish Government, following the recent introduction of a minimum floor price on alcohol in Scotland [39]. Costs were applied to the total number of liquor retailers and liquor venues in Australia, determined through available IBISWorld industry data [40]. For liquor retail stores, we based resource use on Scottish Government estimates [39] using Australian 2010 wage earnings data [41]. This was costed as a mean of 16 hours’ of staff time per store to implement changes (e.g., replace shelf tags, signage, and brief staff on the incoming policy change). Costs to other liquor venues (e.g., hotels, pubs) were calculated at a mean of 2 hours’ time to brief staff and update their point of sale systems. It was assumed that there would be no additional cost associated with changes to menus and signage, given that these are likely to be changed regularly as a normal course of business and so the intervention would not add additional costs given that there would be a substantial notice period before changes took effect. Long term costs to industry (e.g., through a drop in sales) were not included in the analyses.

#### 2.4.3. Changes to Expenditure and Tax Revenue

Population alcohol expenditure was calculated for each alcohol type using 2011–2012 AHS alcohol intake data [9] and 2013 alcohol price estimates [21]. Certain drinks types including cider, mixed drinks (homemade), liqueurs, and cocktails were not included in the change in expenditure and tax revenue analysis, as price data was not available for these alcohol categories. However, consumption data from these categories were included in the overall cost-effectiveness analysis. For the uniform volumetric tax, baseline tax rates (2013) were used to determine the price of various alcoholic beverages post-intervention, along with the amount of tax revenue generated post-Intervention 1. Expenditure and tax revenue data were deflated to 2010 values to reflect the 2010 population.

#### 2.4.4. Cost-Effectiveness Modeling

A limited societal perspective was adopted for costings and calculation of benefits accrued by key stakeholders (both government and non-government). However, due to a lack of data availability, some downstream impacts and indirect costs such as reductions in industry revenue for the volumetric tax intervention and increases in industry revenue for the minimum floor price were not captured.

The time horizon for the evaluation was the lifetime of the 2010 Australian population (or 100 years). A 3% discount factor was adopted for all costs and benefits [42]. Modeling was undertaken in Excel 2013 and second order (parameter) uncertainty analyses were undertaken by applying the Monte-Carlo simulation using the Excel add-in software, Ersatz (version 1.35) [43]. All results were reported in mean values with 95% UIs. 

The mean incremental net costs (intervention costs minus health care cost savings) of the intervention in comparison to the base case scenario were divided by the mean incremental health benefits (HALYs) to calculate an incremental cost-effectiveness ratio (ICER). The intervention was deemed cost-effective if the ICER was less than the commonly-accepted willingness to pay the threshold for Australia (AUD50,000 per HALY gained) [44].

## 3. Results

### 3.1. Changes in Price

The types of alcoholic drinks used in this analysis, their mean energy content, and the prices and tax rates at the baseline and in each intervention scenario are shown in Table 1. 

Both interventions increased the price of all alcohol types, but by varying amounts. The largest price increase was seen for off-premises cask wine (uniform volumetric tax: +157%; minimum floor price: +168%), followed by off-premises wine (uniform volumetric tax: +43%; minimum floor price: +14%), and off-premises regular strength beer (uniform volumetric tax: +39%; minimum floor price: +8%).

### 3.2. Intervention Effectiveness Results 

Estimated alcoholic drink consumption at the baseline and under each intervention scenario are shown in Table 2, along with estimated changes in kilojoule intake, body weight, and BMI. For further details on alcoholic drink consumption, kilojoule intake, and body weight change for each age and sex group, refer to Table S1. For details on the change in consumption by alcohol type, refer to Table S2.

Both interventions were estimated to have substantial impacts on mean alcohol intake and body weight across the Australian population aged 15 years and over. Across males and females, the most significant reductions in alcohol intake and body weight were seen for middle aged and older adults, particularly those aged 65−74 years. 

Introducing a uniform volumetric tax was estimated to reduce the mean intake of all alcohol by 20.7% overall (95% UI: 20.2 to 21.1), while introducing a minimum floor price was estimated to reduce the mean intake of all alcohol by 9.2% overall (95% UI: 8.8 to 9.5). For both interventions, the largest decrease in the consumption of alcohol was seen for off-premises cask wine, followed by off-premises wine. For the most part, the consumption of on-premises alcoholic beverages substantially increased or remained unchanged across alcohol types. This was primarily due to the cross-price elasticity of the different alcohol categories. 

Introducing a uniform volumetric tax was estimated to lead to a −0.90 kg (95% UI: −0.84 to −0.96) weighted average change in weight and a −0.34 kg/m^2^ (95% UI: −0.32 to −0.36) change in BMI. Introducing a minimum floor price corresponded to a −0.45 kg (95% UI: −0.42 to −0.48) weighted average change in weight and a −0.19 kg/m^2^ (95% UI: −0.17 to −0.20) change in BMI. The effect size was slightly greater for males than for females across both interventions, largely due to the higher levels of alcohol consumption among males. 

### 3.3. Cost-Effectiveness Results

**Figure 1:**
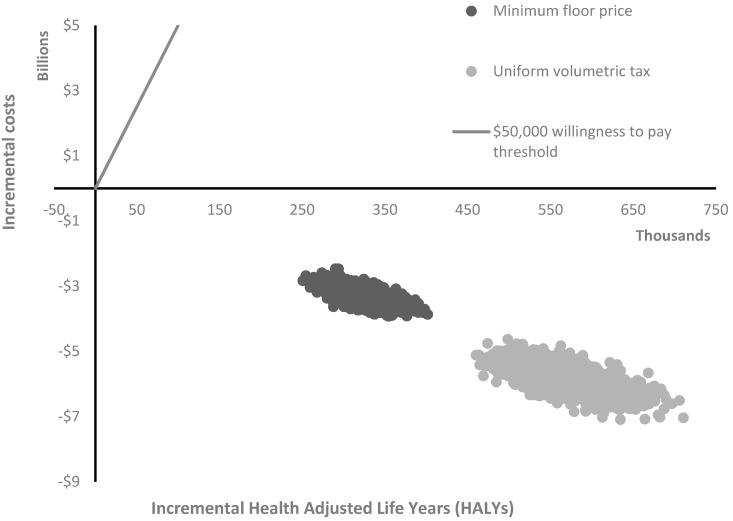
Cost effectiveness plane.

The Australian population aged 15 years and over was expected to gain 566,648 obesity-related HALYs (95% UI: 497,431 to 647,262) from the introduction of a uniform volumetric tax, and 317,653 obesity-related HALYs (95% UI: 276,334 to 361,573) from the introduction of a minimum floor price (refer to Table 3). Both interventions resulted in slightly higher HALY gains for males (universal volumetric tax: 336,862; 95% UI: 286,706 to 395,302; minimum floor price: 159,844; 95% UI: 135,384 to 188,345) compared to females (universal volumetric tax: 229,786; 95% UI: 192,014 to 272,287; minimum floor price: 157,809; 95% UI: 131,304 to 213,704). All ICER iterations of the model for both interventions were dominant, resulting in health benefits and cost savings (refer to Figure 1). Introducing a uniform volumetric tax would result in $5.8 billion (B) (95% UI: $5.1 B to $6.6B) in cost savings to the healthcare system with intervention costs of $24 million (M) (95% UI: $23M to $26M). Introducing a floor price would result in $3.3B (95% UI: $2.9B to $3.7B) in cost savings to the healthcare system, with intervention costs expected to be $30M (95% UI: $26M to $36M) (refer to Table 3). These costs relate primarily to the first year post intervention. A total of 87% and 89% of the intervention costs were borne by government for the uniform volumetric tax and minimum floor price, respectively. For a detailed breakdown of unit costs, resource use, and costs to government and industry, see Tables S3.1–3.3.

### 3.4. Impact on Expenditure and Tax Revenue 

Total population expenditure on alcohol increased post-intervention, with a higher increase seen for the uniform volumetric tax. A uniform volumetric tax was estimated to generate an additional $2.7B in tax revenue for government in the first year of the intervention, equivalent to a 114% relative increase in current (2013) taxes from alcohol. A minimum floor price was expected to generate additional industry revenue, however, this amount was not estimated in our model. For further details on expenditure and tax revenue at the baseline and post-intervention, see Table S4.

### 3.5. Sugar Sweetened Beverage (SSB) Substitution Analysis

After accounting for substitution of alcoholic beverages with full sugar SSBs (see Table S5), both interventions were found to result in net health losses. In this scenario, the Australian population was expected to lose 512,422 HALYs (95% UI: 448,487 to 584,831) from the introduction of a uniform volumetric tax on alcohol, and lose 275,526 HALYs (95% UI: 238,941 to 314,562) from a minimum floor price on alcohol. 

## 4. Discussion

This study has demonstrated that increasing the price of alcohol is likely to be a cost-effective intervention for obesity prevention in Australia, provided consumers substitute alcoholic beverages with low or no kilojoule beverage alternatives. Both a uniform volumetric tax and a minimum floor price would lead to significant reductions in body weight and HALY gains; however, a uniform volumetric tax would have a more substantial effect, primarily due to it increasing the price of all types of alcohol to some extent. 

To date, there have been no other studies that have quantitatively modeled the effects on obesity of fiscal interventions related to alcohol. However, our results are in line with other research that has investigated the effect of alcohol related pricing interventions on alcohol consumption and non-obesity related health outcomes. In Australia, VicHealth conducted a cost-effectiveness analysis in 2011 that estimated the effect of a volumetric tax applied to all alcohol (equivalent to a 10% increase in the 2010 tax rate applied to off-premises spirits). Their results indicated that this scenario would lead to a 10.6% decrease in overall consumption of alcohol, 220,000 disability adjusted life years (DALYs) averted (based on preventing alcohol related harm, not including obesity) and $3.2B in cost savings [16]. Another Australian study that modeled the impact of a volumetric alcohol taxation scenario, applying a tax rate equal to that of spirits, indicated that this could lead to a 24% decrease in consumption of pure alcohol and 170,000 DALYs averted (non-obesity related) [15]. Research from England has shown that a floor price on alcohol of 70 cents GBP (approximately AUD1.30) could lead to an 18% mean reduction in consumption of alcohol [45], and would be an effective measure for reducing alcohol consumption in the English population. In Scotland, the recent introduction of a floor price of 50 cents GPD (93 cents AUD) was previously estimated to reduce consumption by up to 7% for harmful drinkers [39]. The results from these studies are similar to our findings, which estimated the mean reduction in alcohol consumption post-intervention to be around 20.7% and 9.2% for a uniform volumetric tax and a minimum floor price, respectively. The variation across jurisdictions in relation to the change in alcohol consumption post-intervention is likely to be due to differences in alcohol intake between countries, alcohol pricing arrangements, and the variability in price elasticity estimates and modeling methods used across studies. 

The results of this paper are consistent with other analyses showing that targeted taxation of food and beverages in Australia can be cost-effective for obesity prevention. For example, an analysis of the potential cost-effectiveness of a 20% SSB tax in Australia, using equivalent methods and the same model as used in this paper, found it was also likely to be dominant. However, the HALYs gains and cost offsets were more than double for a uniform volumetric tax on alcohol, compared to a 20% SSB tax [27]. This is largely because energy intake from alcohol is higher than from SSBs in the Australian population, and alcohol is primarily consumed by middle aged groups who are at higher risk of developing chronic diseases associated with obesity. 

Our study did not consider the effect of alcohol pricing interventions on different types of drinkers (e.g., low, moderate, and heavy consumers of alcohol) or income groups in the population, which is a limitation of this study. A potential argument against increasing the price of alcohol is that this policy intervention may place a disproportionate financial burden on low-and-moderate consumers of alcohol as well as low-income consumers. However, a recent study investigating the financial impacts of a minimum unit pricing policy (AUD$2.00 per standard drink) found that the additional tax cost placed on low-and-moderate consumers of alcohol was minimal, and did not lead to a disproportionate burden when compared to heavy consumers of alcohol [46]. In terms of the impact of these interventions on different income groups, previous research has indicated that both a minimum floor price and a uniform volumetric tax are likely to be somewhat regressive in nature [47]. However, lower income groups are also likely to experience greater health benefits from alcohol pricing interventions [48]. Recent modeling of a SSB tax in the Australian population demonstrated that an SSB tax is likely to result in the most health gains for the most disadvantaged groups in the population, and the difference in out-of-pocket costs between advantaged and disadvantaged groups would be minimal [32]. In the case of a uniform volumetric tax on alcohol, to mitigate some of the regressive aspects of the tax, generated revenue could be used to fund preventive health initiatives and programs in the community, although this was not investigated as part of the current study. 

A recent study has suggested that taxation of SSBs may be correlated with changes in the demand for alcohol, with the impact varying based on the type of alcohol [49]. To address potential substitution from alcoholic beverages to non-alcoholic beverages in response to the intervention, our study included a scenario that assumed substitution to full sugar SSBs post intervention. We did not take into account potential substitution to foods or other non-alcoholic drinks such as juice or flavored milks, due to a lack of available data on the substitution effects of alcohol to these products. The results of this scenario analysis showed that both a uniform volumetric tax and minimum floor price would likely result in overall health losses and would not be cost-effective compared to the no intervention comparator. These findings are not surprising given the assumption that a serving of alcohol (e.g., 150 mL glass of wine or 30 mL shot of spirits) would be substituted with a 375 mL can of full sugar SSB that has a high kilojoule content. Whilst more research is needed to understand potential substitution behavior, this analysis indicates the importance of considering unintended effects resulting from an increase in the price of alcohol. Consideration needs to be given to combining increases in alcohol taxes with taxes on SSBs. In addition, public health campaigns to increase awareness of alcohol pricing interventions (such as the one assessed in this study) could include messaging around encouraging consumers to switch alcohol with low or no-kilojoule beverage alternatives. 

International and local evidence suggests that acceptability from industry, government, and the public is likely to be a key barrier to implementation of any price increase on alcohol [50,51]. The alcohol industry is predominantly opposed to fiscal interventions [52] and governments will likely face ongoing resistance from industry. Research involving alcohol industry representatives in Australia and Scotland has suggested that certain sectors such as retailers may be somewhat supportive of a floor price on alcohol [39,53], due to the potential for revenue. In Scotland, the Sheffield study estimated that industry revenue from the introduction of a 50 p minimum unit price scenario would be equivalent to around £34 million (AUD 61 million) [39]. Revenue increases for the alcohol industry has also been raised as a concern by opponents of minimum pricing [39,54]. It will be important for governments to weigh up these concerns whilst monitoring the recent implementation of the minimum floor price in the Northern Territory. Policy makers may find a uniform volumetric tax intervention favorable due to the increase in revenue that would be generated from the change in tax structure. Our study estimated that an additional $2.7B in tax revenue would be raised at a federal level in the first year following the introduction of a uniform volumetric tax. Research indicates that the transparent use of tax revenue and diversion of tax revenue into public health initiatives is likely to increase community acceptability and public support [55,56]. As such, the framing of an alcohol tax, along with associated public health advocacy, will need to be carefully considered. The Australian government is currently considering policy options for kilojoule labeling of alcoholic beverages [57] in response to the 2011 Labeling Logic review, which recommended alcoholic beverages display energy labels to align with requirements for other foods and beverages [58]. If kilojoule labeling is implemented on alcoholic drinks, this will signify an important first step in acknowledging the kilojoule contribution of alcohol in the Australian diet, and may help consumers to better understand and choose lower kilojoule alcohol options.

In this study, we assumed that a reduction in kilojoules from alcoholic beverages would have an equivalent effect on weight as a reduction in kilojoules from food or non-alcoholic beverages. There is good evidence to suggest that alcohol consumption increases overall energy intake. A recent systematic review of 22 studies found that alcohol intake was significantly associated with an increase in food energy intake and total energy intake, and that usual dietary intake did not decrease to compensate for energy consumed from alcohol [11]. Nevertheless, studies that have examined the relationship between alcohol and weight gain have shown mixed and sometimes conflicting results for different types of drinkers [3,59,60,61,62]. The inconsistencies in the evidence base are likely at least partly due to a range of confounders associated with alcohol consumption and obesity including educational status, physical activity level, and income [59,63]. For example, higher frequency drinkers may be more likely to have professional jobs and a higher income, which could lead them to exercise more frequently or have more nutritional diets [59,63]. Differences in results could also be due to the use of relatively weak study designs such as cross-sectional studies that use self-reported BMI and alcohol consumption, which may lead to difficulties in isolating associations [61]. Further understanding of the interaction between alcohol consumption and weight gain, potentially using strengthened study designs, may help to clarify some of the uncertainties in the evidence base. Additionally, it will be important for future modeling studies to look at how the effectiveness (from an obesity prevention perspective) of alcohol pricing policies may differ across different types of drinkers (e.g., low, moderate, and heavy) in the population.

This study was the first of its kind to quantitatively model the effects of fiscal alcohol interventions on obesity specifically and provides a novel contribution to the literature. A strength of this study was that it used Australian specific data on alcohol intake and relevant price elasticity estimates to calculate the change in the consumption of alcohol and effect on weight in the Australian population, and accounted for a wide range of alcohol categories including off-premises and on-premises alcohol.

A limitation of this study was that we were restricted to using available 2013 data on alcohol price, proportion of alcohol sold under $1.30, and tax rates to calculate the change in price post-intervention. We therefore made the assumption that the 2013 price data and subsequent changes to alcohol consumption would apply to the 2010 Australian population. We were limited to using Scottish data to estimate resource use for industry costs in response to the two interventions, due to a lack of available data for Australia. We also did not take into account industry revenue from the introduction of a minimum unit floor price as part of a societal perspective, however, we expect that industry profits would benefit from the introduction of a floor price. In Scotland, the Sheffield study estimated that industry revenue from the introduction of a 50 p minimum unit price scenario would be equivalent to around AUD 61 million [39]. We were restricted to using self-reported alcohol consumption data, which are widely known to be underreported. Future research could consider incorporating alcohol purchasing data or online sales data (e.g., through Euromonitor) with health survey data to more accurately determine alcohol intake in the population. Our study made several price elasticity assumptions: the price elasticity of bottled wine was applied to all wine categories; the price elasticity of spirits was applied to liqueurs, cocktails, mixed drinks; the price elasticity of regular beer was applied to cider. Due to the large increase in the price of cask wine, consumption of cask wine was estimated to fall to 0 mL/day post-intervention based on the price elasticities used, which is unlikely to represent the actual change in consumption. We assumed that changes in alcohol consumption and associated weight reductions would remain in place over the life time of the cohort. This is consistent with modeling conducted for other taxation interventions associated with obesity prevention [32]; however, the impact of the intervention over time needs to be further examined. We also did not consider health benefits outside of those related to obesity and therefore underestimated the full health impact of reduced alcohol consumption from alcohol related disease and injuries [64]. Future studies should consider the health benefits from both reductions in BMI-related diseases and alcohol-related harms. 

## 5. Conclusions

This study was the first of its kind to model the obesity-related effects of a uniform volumetric tax and minimum unit floor price on alcohol at the population level in Australia. Our analysis indicated that both approaches would result in substantial health gains and cost-savings for the Australian population, provided consumers substitute alcoholic beverages with low or no kilojoule beverage alternatives. Further research is needed to better understand the impact of alcohol pricing policies on obesity-related health outcomes. Overhauling the current taxation system on alcohol and replacing it with either a uniform volumetric tax or a minimum floor price will take strong leadership from policy makers, and will likely be met by significant pushback from the alcohol industry and the public. 

## Figures and Tables

**Table 1:** Alcohol categories, tax rate, and price changes post-intervention.

Alcohol Type Included in Analysis ^1^	Mean Energy Content (kJ per 100 mL (95% UI))	Baseline Mean Price ^2^ per Standard Drink $ (95% CI) [21]	Baseline Tax Rate ^2^ per Standard Drink $ [20]	Baseline Proportion of Alcohol Sold under $1.30	Post-Uniform Volumetric Tax (Intervention 1)			Post-Minimum Floor Price (Intervention 2) ^3^		Price Elasticity Applied [21]
					Tax Rate per Standard Drink	Mean Price per Standard Drink $ (95% CI)	Change in Price per Standard Drink $ (%)	Mean Price per Standard Drink $ (95% CI)	Change in Price per Standard Drink $ (%)	
Off-premises beer, full strength (>3.5% alcohol)	158 (136–223) kJ	1.57 (1.51–1.63)	0.46	39.50%	1.07	2.18 (2.12–2.24)	0.61 (+39%)	1.70 (1.64–1.76)	0.13 (+8%)	Off-Regular beer
On-premises beer, full strength (>3.5% alcohol)	158 (136–223) kJ	4.41 (4.24–4.58)	0.31	1.00%	1.07	5.17 (5.00–5.34)	0.76 (+17%)	4.43 (4.26–4.60)	0.02 (+0.4%)	On-Regular beer
Off-premises beer, mid-light (1.15–3.5% alcohol)	111 (103–120) kJ	2.31 (2.01–2.62)	0.40	3.04%	1.07	2.98 (2.68–3.29)	0.67 (+29%)	2.33 (2.03–2.64)	0.02 (+0.8%)	Off-Mid-strength beer
On-premises beer, mid-light (1.15–3.5% alcohol)	111 (103–120) kJ	5.99 (5.62–6.37	0.22	0.00%	1.07	6.84 (6.47–7.22)	0.85 (+14%)	5.99 (5.62–6.37)	0.00 (0%)	On-Mid-strength beer
Off-premises wine (including red and white)	307 (268–338) kJ	1.96 (1.78–2.13)	0.22	37.50%	1.07	2.81 (2.63–2.98)	0.85 (+43%)	2.24 (2.06–2.41)	0.28 (+14%)	Off-Bottled wine
On-premises wine (including red and white)	307 (268–338) kJ	6.25 (5.80–6.71)	0.22	0.88%	1.07	7.10 (6.65–7.56)	0.85 (+14%)	6.27 (5.82–6.73)	0.02 (+0.3%)	On-Bottled wine
Off-premises cask wine	307 (268–338) kJ	0.65 (0.47–0.83)	0.05	98.30%	1.07	1.67 (1.49–1.85)	1.02 (+157%)	1.74 (1.56–1.92)	1.09 (+168%)	Off-Cask wine
Off-premises spirits	893 (886–912) kJ	1.67 (1.52–1.83)	0.97	24.80%	1.07	1.77 (1.62–1.93)	0.10 (+6%)	1.75 (1.60–1.91)	0.08 (+5%)	Off-Spirits
On-premises spirits	893 (886–912) kJ	5.34 (4.76–5.91)	0.97	1.37%	1.07	5.44 (4.86–6.01)	0.10 (+2%)	5.38 (4.80–5.95)	0.04 (+0.8%)	On-Spirits
Off-premises pre-mixed drinks, commercial	252 (234–281) kJ	2.79 (2.46–3.12)	0.97	0.51%	1.07	2.89 (2.56–3.22)	0.10 (+4%)	2.80 (2.47–3.13)	0.01 (+0.3%)	Off-RTD’s
On-premises pre-mixed drinks	238 (109–446) kJ	6.35 (5.90–6.79)	0.97	0.09%	1.07	6.45 (6.00–6.89)	0.10 (+2%)	6.35 (5.90–6.79)	0.00 (0%)	On-RTD’s

CI: Confidence Interval. UI: Uncertainty Interval. RTD: Ready to drink. ^1^ Cider, mixed drinks (homemade), liqueurs and cocktails are not included in this analysis as the price data were not available for these alcohol categories. ^2^ $ AUD 2013. ^3^ Tax rate remains unchanged for this intervention.

**Table 2:** Change in the consumption of alcoholic drinks, kilojoule intake, body weight, and BMI post-intervention (mean values, weighted by population size for five year age groups > 15 years).

Baseline Consumption of Alcoholic Drinks, mL/day per Person (95% UI)	182.0 (172.2 to 192.2)	
	Uniform Volumetric Tax	Minimum Floor Price
Change in daily consumption of alcoholic drinks, mL/day per person (95% UI)	−37.6 (−35.7 to −39.6)	−16.7 (−15.9 to −17.7)
Percentage change in consumption of alcoholic drinks	−20.7% (−20.2 to −21.1)	−9.2% (−8.9 to −9.6)
Change in kilojoule intake, kJ/day per person (95% UI)	−90.0 (−84.1 to −96.2)	−44.8 (−41.9 to −48.0)
Change in body weight (kg/person)	−0.90 (−0.84 to −0.96)	−0.45 (−0.42 to −0.48)
Change in BMI (kg/m^2^)	−0.34 (−0.32 to −0.36)	−0.19 (−0.17 to −0.20)

kJ: Kilojoule. kg: kilogram. m^2^: meters squared. mL: milliliter. BMI: body mass index. UI: Uncertainty interval.

**Table 3:** Cost-effectiveness and health gains.

Cost Effectiveness and Health Gains	Uniform Volumetric Tax Mean (95% UI)	Minimum Floor Price Mean (95% UI)
Total intervention costs	$24M ($23M to $26M)	$30M ($26M to $36M)
Total healthcare cost savings	$5.8B ($5.1B to $6.6B)	$3.3B ($2.9B to $3.7B)
Net costs *	−$5.8B (−$6.6B to −$5.1B)	−$3.3B (−$3.7B to −$2.8B)
Total HALYs gained	566,648 (497,431 to 647,262)	317,653 (276,334 to 361,573)
ICER ($/HALY gained)	Dominant (dominant to dominant) **	Dominant (dominant to dominant) **
Probability of being cost effective ***	100%	100%

B: billion. M: million. UI: uncertainty interval. $: AUD 2010. HALYs: Health Adjusted Life Years. ICER: incremental cost-effectiveness ratio. * Negative costs are cost savings ** Dominant: the intervention is both cost-saving and improves health. Negative total net costs equate to cost savings. *** The willingness-to-pay threshold for this analysis is $50,000 per health adjusted life year gained.

## Supplementary Materials

The following are available online at https://www.mdpi.com/2072-6643/12/3/603/s1, Table S1: Baseline and post-intervention alcohol consumption, kilojoule intake and weight change; Table S2: Baseline and post-intervention alcohol intake, by alcohol type; Table S3.1: Unit costs and resource use; Table S3.2: Input parameters; Table S3.3: Intervention costs by component and sector; Table S4: Baseline and post-intervention expenditure on alcohol and tax revenue, by alcohol type; Table S5: SSB substitution analysis.
