# Supplementary material for: Increasing the Price of Alcohol as an Obesity Prevention Measure: The Potential Cost-Effectiveness of Introducing a Uniform Volumetric Tax and a Minimum Floor Price on Alcohol in Australia
Source: Nutrients. 2020 Feb 26;12(3):603. doi: 10.3390/nu12030603 (PMC7146351; doi:10.3390/nu12030603)
Supplement: Supplementary file 1 [file nutrients-12-00603-s001.zip › S1 Table - Change in consumption, kJ, body weight.docx]

#### **S1 Table: Baseline and post-intervention alcohol consumption, kilojoule intake and weight change (for age and sex group)**

| Baseline | | | | | | | | | | | | | | | | | | |
| --- | --- | --- | --- | --- | --- | --- | --- | --- | --- | --- | --- | --- | --- | --- | --- | --- | --- | --- |
| Consumption of alcoholic drinks | | | | | | | | | | | | | | | | | | |
| Age | **15-19** | | **20-24** | | **25-34** | | **35-44** | | **45-54** | | **55-64** | | **65-74** | | **75-100** | | | **Mean (weighted by population size >15 years)**  **(95% UI)** |
| Sex | **Male** | **Female** | **Male** | **Female** | **Male** | **Female** | **Male** | **Female** | **Male** | **Female** | **Male** | **Female** | **Male** | **Female** | **Male** | **Female** | |  |
| Mean daily consumption (ml/day per person) | 81.1 | 16.4 | 264.0 | 100.7 | 343.9 | 93.7 | 351.5 | 131.8 | 425.2 | 136.7 | 419.2 | 137.2 | 370.1 | 152.4 | 178.5 | 86.3 | | 182.0  (172.2 to 192.2) |
| Uniform volumetric tax | | | | | | | | | | | | | | | | | | |
| Change in consumption of alcoholic drinks | | | | | | | | | | | | | | | | | | |
| Age | **15-19** | | **20-24** | | **25-34** | | **35-44** | | **45-54** | | **55-64** | | **65-74** | | **75-100** | | | **Mean (weighted by population size >15 years)**  **(95% UI)** |
| Sex | **Male** | **Female** | **Male** | **Female** | **Male** | **Female** | **Male** | **Female** | **Male** | **Female** | **Male** | **Female** | **Male** | **Female** | **Male** | **Female** | |  |
| Change in daily consumption (ml/day per person) | -13.3 | -2.8 | -41.2 | -20.0 | -56.0 | -19.3 | -62.4 | -30.5 | -83.3 | -34.4 | -80.4 | -40.6 | -72.0 | -48.2 | -38.3 | -22.0 | | -37.6  (-35.7 to -39.6) |
| Overall percentage change in consumption | -16.4% | -16.9% | -15.6% | -19.8% | -16.3% | -20.6% | -17.8% | -23.1% | -19.6% | -25.1% | -19.2% | -29.6% | -19.5% | -31.6% | -21.5% | -25.8% | | -20.7%  (-20.2 to -21.1) |
| Change in kilojoule intake (kJ/day per person) | -27.6 | -6.8 | -71.8 | -55.6 | -107.3 | -47.7 | -128.6 | -84.0 | -185.3 | -97.4 | -181.7 | -120.7 | -171.1 | -143.0 | -98.1 | -65.5 | | -90.0  (-84.1 to -96.2) |
| Change in Body weight | | | | | | | | | | | | | | | | | | |
| Change in body weight (kg/person) | -0.26 | -0.07 | -0.72 | -0.56 | -1.1 | -0.48 | -1.3 | -0.84 | -1.9 | -0.97 | -1.8 | -1.2 | -1.7 | -1.4 | -1.0 | -0.7 | | -0.90  (-0.84 to -0.96) |
| Weighted average change in BMI (kg/m^2^) |  | | | | | | | | | | | | | | | | | -0.34  (-0.32 to -0.36) |
| Minimum floor price | | | | | | | | | | | | | | | | | | |
| Change in consumption of alcoholic drinks | | | | | | | | | | | | | | | | | | |
| Age | **15-19** | | **20-24** | | **25-34** | | **35-44** | | **45-54** | | **55-64** | | **65-74** | | **75-100** | | **Mean (weighted by population size >15 years)**  **(95% UI)** | |
| Sex | **Male** | **Female** | **Male** | **Female** | **Male** | **Female** | **Male** | **Female** | **Male** | **Female** | **Male** | **Female** | **Male** | **Female** | **Male** | **Female** |  | |
| Change in daily consumption (ml/day per person) | -4.5 | -1.1 | -14.8 | -10.0 | -21.3 | -11.3 | -25.4 | -19.3 | -36.0 | -22.6 | -34.7 | -26.7 | -31.7 | -31.4 | -17.9 | -14.5 | -16.7  (-15.9 to -17.7) | |
| Overall percentage change in consumption | -5.6% | -6.5% | -5.6% | -9.9% | -6.2% | -12.0% | -7.2% | -14.6% | -8.5% | -16.5% | -8.3% | -19.5% | -8.6% | -20.6% | -10.0% | -16.8% | - 9.2%  (-8.9 to -9.6) | |
| Change in kilojoule intake (kJ/day per person) | -9.4 | -2.8 | -26.4 | -29.8 | -43.3 | -40.0 | -56.4 | -56.5 | -86.5 | -67.2 | -84.5 | -81.4 | -80.8 | -95.5 | -48.9 | -44.8 | -44.8  (-41.9 to -48.0) | |
| Change in Body weight | | | | | | | | | | | | | | | | | | |
| Change in weight (kg/person) | -0.09 | -0.03 | -0.26 | -0.30 | -0.43 | -0.31 | -0.56 | -0.57 | -0.86 | -0.67 | -0.84 | -0.81 | -0.81 | -0.96 | -0.49 | -0.45 | -0.45  (-0.42 to -0.48) | |
| Weighted average change in BMI (kg/m^2^) |  | | | | | | | | | | | | | | | | -0.19  (-0.17 to -0.20) | |
