# Supplementary material for: Increasing the Price of Alcohol as an Obesity Prevention Measure: The Potential Cost-Effectiveness of Introducing a Uniform Volumetric Tax and a Minimum Floor Price on Alcohol in Australia
Source: Nutrients. 2020 Feb 26;12(3):603. doi: 10.3390/nu12030603 (PMC7146351; doi:10.3390/nu12030603)
Supplement: Supplementary file 1 [file nutrients-12-00603-s001.zip › S2 Table - Consumption by alcohol type.docx]

#### **S2 Table: Baseline and post-intervention alcohol intake, by alcohol type**

| **Alcohol type included in analysis** | **Baseline** | **Post-uniform volumetric tax**  **(Intervention 1)** | | **Post-minimum floor price**  **(Intervention 2)** | |
| --- | --- | --- | --- | --- | --- |
|  | **Population consumption**  **(ml/day)** | **Population consumption (ml/day)** | **Change in consumption**  **ml/day (%)** | **Population consumption (ml/day)** | **Change in consumption**  **ml/day (%)** |
| Off-premises beer, full strength (> 3.5% alcohol) | 64.2 | 47.8 | -16.4 (-26%) | 58.3 | -5.9 (-9%) |
| On-premises beer, full strength (> 3.5% alcohol) | 18.2 | 21.1 | 2.9 (16%) | 19.2 | 1.0 (5%) |
| Off-premises beer, mid-light (1.15- 3.5% alcohol) | 20.3 | 18.0 | -2.3 (-11%) | 20.7 | 0.4 (2%) |
| On-premises beer, mid-light (1.15- 3.5% alcohol) | 5.8 | 5.7 | -0.1 (-2%) | 5.8 | 0.0 (0%) |
| Off-premises wine (including red and white) | 33 | 19.6 | -13.4 (-41%) | 28.3 | -4.7 (-14%) |
| On-premises wine (including red and white) | 10.2 | 12.2 | 2.0 (20%) | 10.9 | 0.7 (7%) |
| Off-premises cask wine | 7.0 | 0.0 | -7.0 (-100%) | 0.0 | -7.0 (-100%) |
| Off-premises spirits | 2.1 | 2.0 | -0.1 (-5%) | 2.0 | -0.1 (-5%) |
| On-premises spirits | 0.5 | 0.5 | 0.0 (0%) | 0.5 | 0.0 (0%) |
| Off-premises pre-mixed drinks, commercial | 9.6 | 7.5 | -2.1 (-22%) | 8.9 | -0.7 (-7%) |
| On-premises pre-mixed drinks, commercial | 1.6 | 1.6 | 0.0 (0%) | 1.6 | 0.0 (0%) |
| Off-premises mixed drinks, homemade | 2.7 | 2.6 | -0.1 (-4%) | 2.6 | -0.1 (-4%) |
| On-premises mixed drinks, homemade | 0.6 | 0.6 | 0.0 (0%) | 0.6 | 0.0 (0%) |
| Off-premises cider | 3.0 | 2.2 | -0.8 (-27%) | 2.7 | -0.3 (-10%) |
| On-premises cider | 0.8 | 1.0 | 0.2 (25%) | 0.9 | 0.1 (13%) |
| Off-premises liqueurs | 0.3 | 0.3 | 0.0 (0%) | 0.3 | 0.0 (0%) |
| On-premises liqueurs | 0.1 | 0.1 | 0.0 (0%) | 0.1 | 0.0 (0%) |
| Off-premises cocktails | 0.5 | 0.5 | 0.0 (0%) | 0.5 | 0.0 (0%) |
| On-premises cocktails | 0.1 | 0.1 | 0.0 (0%) | 0.1 | 0.0 (0%) |
