# Supplementary material for: Increasing the Price of Alcohol as an Obesity Prevention Measure: The Potential Cost-Effectiveness of Introducing a Uniform Volumetric Tax and a Minimum Floor Price on Alcohol in Australia
Source: Nutrients. 2020 Feb 26;12(3):603. doi: 10.3390/nu12030603 (PMC7146351; doi:10.3390/nu12030603)
Supplement: Supplementary file 1 [file nutrients-12-00603-s001.zip › S3.1 Table - Unit costs, resource use.docx]

#### **S3.1 Table: Unit costs and resource use**

| **Cost item** | **Unit cost** | **Uncertainty distribution** | **Resource use** | **Assumption** | **Source** |
| --- | --- | --- | --- | --- | --- |
| ***Cost to government*** | | | |  |  |
| Cost of passing legislation | $1,090,766 | Gamma  (SE: $77,497) | One off cost | Cost year 1  *(Intervention 1 and 2)* | Source: Lal et al. (25) |
| Tax audit cost | | | | | |
| Accountant salary (per week) | $1,635 | Gamma  (SE: $63) | 0.32FTE government staff (per million people^1^) | Cost year 1  *(Intervention 1)* | Source: Lal et al. (25), Long et al. (26) |
| Field audit salary (per week) | $1,635 | Gamma  (SE: $63) | 0.30FTE government staff (per million people) | Cost year 1  *(Intervention 1)* | Source: Lal et al. (25), Long et al. (26) |
| Field audit direct costs | $13,780 | Gamma  (SE: $1,756) | Per million people | Cost year 1  *(Intervention 1)* | Source: Lal et al. (25), Long et al. (26) |
| Tax certification system operating costs | $17,883 | Gamma  (SE: $2,684) | Per million people | Cost year 1  *(Intervention 1)* | Source: Lal et al. (25), Long et al. (26) |
| Government staff cost | | | | | |
| Government staff cost for ‘high level advice’ to major liquor chains (per week) | $1,555 | Gamma  (SE: $44.8) | 0.032FTE government staff per major liquor chain advised (national level) | Cost year 1  *(Intervention 2)* | Source: Australian Bureau of Statistics (Professional, Scientific and Technical Services earnings) (31) |
| Government cost for monitoring compliance (per week) | $1,555 | Gamma  (SE: $44.8) | 0.006FTE government staff per store monitored. 7% of liquor retail stores monitored | Annual ongoing cost  *(Intervention 1 and 2)* | Source: NSW Food Authority 2012 (26) |
| Consumer education campaign cost | | | | | |
| Government staff cost for consumer education campaign coordination (per week) | $1,555 | Gamma  (SE: $44.8) | 2.2FTE government staff | Cost year 1  *(Intervention 1 and 2)* | Source: NSW Food Authority 2012 (26) |
| Government campaign cost, outsourced work to run consumer education campaign (one off cost) | $1,108,249 | UI n/a | Cost per state/territory | Cost year 1  *(Intervention 1 and 2)* | Source: NSW Food Authority 2012 (26) |
| ***Cost to industry*** | | | | | |
| Industry staff cost for liquor venues (per hour) | $27 | Gamma (mean weekly earning $950.6; SE: $19.6) | Assume 2.0 hours (Pert distribution min 1.0, max 3.0) manager staff time for briefing staff members per venue | Cost year 1  *(Intervention 1 and 2)* | Source (earnings): Australian Bureau of Statistics (Retail trade earnings) (31) |
| Industry staff cost for liquor retail stores (per hour) | $27 | Gamma (mean weekly earning $950.6; SE: $19.6) | Assume 16.0 hours (Pert distribution min 8.0, max 24.0) manager staff time to update price tags, update system and brief staff members per venue | Cost year 1  *(Intervention 1 and 2)* | Source (earnings): Australian Bureau of Statistics (Retail trade earnings) (31)  Source (resource use): Scottish Floor Pricing RIS 2018 (29) |

SE: Standard Error. FTE: full time equivalent. NSW: New South Wales. RIS: Regulatory Impact Statement. ^1^ AU population (2010) 15 years and over
