# Supplementary material for: Increasing the Price of Alcohol as an Obesity Prevention Measure: The Potential Cost-Effectiveness of Introducing a Uniform Volumetric Tax and a Minimum Floor Price on Alcohol in Australia
Source: Nutrients. 2020 Feb 26;12(3):603. doi: 10.3390/nu12030603 (PMC7146351; doi:10.3390/nu12030603)
Supplement: Supplementary file 1 [file nutrients-12-00603-s001.zip › S3.2 Table - Input parameters.docx]

| Input parameters | Input amount | Assumption | Source |
| --- | --- | --- | --- |
| Number major liquor chains advised* | 16 | Only the largest liquor chains will require high level advice on implementation of a minimum floor price (new initiative). Does not include major liquor chains that operate exclusively online. | Source: McCusker Centre for Action on Alcohol and Youth, Curtin University 2017 (51) |
| Number of liquor venues | 8437 | Liquor venues includes the total number of bars, clubs, nightclubs, hotels, and resorts in Australia. | Source: IBIS World Industry Statistics – Australia (30) |
| Number of liquor retail stores | 6374 | Liquor retail stores includes the total number of liquor retailers in Australia. | Source: IBIS World Industry Statistics - Australia (30) |
| Number of liquor retail stores monitored | 451 | 7% sample of total number of liquor retail stores require monitoring | Source: NSW food authority 2012 (26) |

#### **S3.2 Table: Input parameters**

*Relevant to Intervention 2 only.
