# Supplementary material for: Increasing the Price of Alcohol as an Obesity Prevention Measure: The Potential Cost-Effectiveness of Introducing a Uniform Volumetric Tax and a Minimum Floor Price on Alcohol in Australia
Source: Nutrients. 2020 Feb 26;12(3):603. doi: 10.3390/nu12030603 (PMC7146351; doi:10.3390/nu12030603)
Supplement: Supplementary file 1 [file nutrients-12-00603-s001.zip › S3.3 Table - Intervention costs.docx]

#### **S3.3 Table: Intervention costs by component and sector**

| **Intervention component costs by sector** | **Intervention cost** | | **Key assumptions** |
| --- | --- | --- | --- |
|  | **Intervention 1: Uniform volumetric tax** | **Intervention 2:**  **Minimum floor price** |  |
| ***Costs to government*** | | | |
| Passing legislation | $1,090,766 ($810,457 to $1,398,519) | $8,715,617 ($8,423,747 to $9,008,027) | Based on estimates of cost of passing legislation in one jurisdiction (25). Intervention 1 implemented at the Federal level; Intervention 2 implemented at the state/territory level. |
| Tax audit cost | $1,782,726 ($1,424,437 to $2,229,261) | n/a | Cost year 1 |
| Monitoring compliance cost | $242,816 ($228,045 to $257,195) | $243,082 ($229,326 to $257,416) | Annual ongoing cost |
| Consumer education campaign | $1,108,249 (UI n/a)  (campaign cost)  $177,804 ($166,988 to $188,333)  (campaign coordination and management) | $1,108,249 (UI n/a)  (campaign cost)  $177,999 ($167,925 to $188,495)  (campaign coordination and management) | Based on campaign at state/territory level. Modelled at national level |
| ‘High level advice’ to major liquor chains | n/a | $41,492 ($39,143 to $43,938) | High level advice applies to minimum floor price only, as this is a new system of pricing (industry familiar with taxation changes) |
| Total cost to government | $21,038,395  ($20,294,138 to $21,777,683) | $26,925,553  ($22,781,188 to $32,326,703) | Includes cost for all states/territories |
| ***Costs to industry*** | | | |
| Cost to liquor retail stores | $2,762,666 ($1,758,812 to $3,763,239) | $2,777,282 ($1,821,374 to $3,743,217) | Includes staff time cost for 6,374 liquor retailers |
| Cost to liquor venues | $456,748 ($292,707 to $621,224) | $456,036 ($290,222 to $622,555) | Includes staff time cost for 8,437 liquor venues |
| Total cost to industry | $3,219,414 ($2,196,247 to $4,256,806) | $3,224,804 ($2,220,972 to $4,245,031) |  |

Note: $: AUD 2010; UI: Uncertainty interval
