# Supplementary material for: Increasing the Price of Alcohol as an Obesity Prevention Measure: The Potential Cost-Effectiveness of Introducing a Uniform Volumetric Tax and a Minimum Floor Price on Alcohol in Australia
Source: Nutrients. 2020 Feb 26;12(3):603. doi: 10.3390/nu12030603 (PMC7146351; doi:10.3390/nu12030603)
Supplement: Supplementary file 1 [file nutrients-12-00603-s001.zip › S4 Table - Expenditure and tax revenue.docx]

#### **S4 Table: Baseline and post intervention expenditure on alcohol and tax revenue, by alcohol type**

| **Alcohol type included in analysis^1^** | **Baseline** | | **Post-uniform volumetric tax (Intervention 1)** | | **Post-minimum floor price (Intervention 2)** | |
| --- | --- | --- | --- | --- | --- | --- |
|  | **Baseline population expenditure (per year)^2^** | **Baseline population tax revenue (per year)** | **Population expenditure (per year)** | **Population tax revenue (for year 1)** | **Population expenditure (per year)** | **Population tax revenue (for year 1)** |
| Off-premises beer, full strength (> 3.5% alcohol) | $2,369,703,665 | $694,308,081 | $2,447,863,066 | $1,201,474,074 | $2,333,280,405 | $630,123,093 |
| On-premises beer, full strength (> 3.5% alcohol) | $1,888,373,048 | $132,742,777 | $2,566,948,441 | $531,263,991 | $2,002,475,438 | $140,162,841 |
| Off-premises beer, mid-light (1.15- 3.5% alcohol) | $680,833,287 | $117,893,210 | $776,686,808 | $279,208,293 | $697,558,677 | $119,789,760 |
| On-premises beer, mid-light (1.15- 3.5% alcohol) | $500,853,185 | $18,395,275 | $566,383,355 | $88,706,007 | $500,888,441 | $18,396,570 |
| Off-premises wine (including red and white) | $4,096,683,428 | $459,831,813 | $3,504,954,293 | $1,334,626,724 | $4,027,970,836 | $395,338,660 |
| On-premises wine (including red and white) | $4,031,016,841 | $141,891,793 | $5,445,275,031 | $820,625,956 | $4,320,581,342 | $151,611,895 |
| Off-premises cask wine | $277,208,448 | $21,323,727 | $0^3^ | $ - | $0 | $ - |
| Off-premises spirits | $699,102,944 | $406,065,782 | $704,777,921 | $426,052,190 | $703,553,469 | $388,999,497 |
| On-premises spirits | $524,365,970 | $95,249,999 | $536,383,925 | $105,501,986 | $530,944,232 | $18,396,570 |
| Off-premises pre-mixed drinks, commercial | $653,064,751 | $227,051,186 | $533,045,171 | $197,355,825 | $610,490,562 | $211,677,889 |
| On-premises pre-mixed drinks, commercial | $243,978,589 | $37,269,170 | $250,109,463 | $41,491,027 | $244,101,546 | $18,771,095 |
| Total | $15,965,184,154 | $2,352,022,812 | $17,332,427,475 | $5,026,306,072 | $15,971,844,948 | $2,093,267,869 |
| **Change in expenditure/tax revenue** |  |  | $1,367,243,320  **(9%)** | $2,674,283,260  **(114%)** | $6,660,793  **(0.04%)** | -$258,754,942  **(-11%)** |

^1^ Cider, mixed drinks (homemade), liqueurs and cocktails are not included in this analysis as 2013 price data was not available for these alcohol categories

^2^ Deflated to $ AUD 2010

^3^ Due to the significant increase in the price of cask wine, consumption of cask wine was estimated to fall to 0ml/day post-intervention, and as such, expenditure was estimated at $0 post-intervention.
