# Supplementary material for: Increasing the Price of Alcohol as an Obesity Prevention Measure: The Potential Cost-Effectiveness of Introducing a Uniform Volumetric Tax and a Minimum Floor Price on Alcohol in Australia
Source: Nutrients. 2020 Feb 26;12(3):603. doi: 10.3390/nu12030603 (PMC7146351; doi:10.3390/nu12030603)
Supplement: Supplementary file 1 [file nutrients-12-00603-s001.zip › S5 Table - SSB substitution analysis.docx]

#### **S5 Table: SSB substitution analysis**

| **Consumption and kilojoule intake** | **Uniform volumetric tax**  **Mean (95% UI)** | **Minimum floor price**  **Mean (95% UI)** |
| --- | --- | --- |
| Mean change in consumption of alcoholic drinks (ml/day per person) | -37.6  (-35.7 to -39.6) | -16.7  (-15.9 to -17.7) |
| Mean change in kilojoule intake from alcoholic drinks (kJ/day per person) | -90.0  (-84.1 to -96.2) | -44.8  (-41.9 to -48.0) |
| Mean kilojoule intake from SSB substitution (kJ/day per person) | 163.4  (155.5 to 171.8) | 81.4  (77.2 to 85.7) |
| Mean change in kilojoule intake, including SSB substitution (kJ/day per person) | 73.4  (67.8 to 79.4) | 36.5  (33.8 to 39.3) |
| Mean change in body weight (kg/person) | 0.73  (0.68 to 0.79) | 0.37  (0.34 to 0.39) |
| **Cost effectiveness and health gains** | **Uniform volumetric tax**  **Mean (95% UI)** | **Minimum floor price**  **Mean (95% UI)** |
| Total intervention costs | $24M ($23M to $26M) | $30M ($26M to $36M) |
| Total healthcare costs | $5.2B ($4.6B to $6.0B) | $2.8B ($2.4B to $3.2B) |
| Net costs | $5.3B ($4.6B to $6.0B) | $2.9B ($2.5B to $3.3B) |
| Total HALYs gained* | -512,422  (-584,831 to -448,487) | -275,526  (-314,562 to -238,941) |
| ICER ($/HALY gained) | Dominated  (dominated to dominated)** | Dominated  (dominated to dominated)** |

SSB: Sugar sweetened beverage. ml: millilitre. kJ: kilojoule. B: billion. M: million. UI: uncertainty interval. $: 2010 Australian dollars. HALY: Health Adjusted Life Years.

^*^ Negative HALYs represent losses to health.

^**^Dominated: the intervention costs more and results in health losses compared to the no intervention comparator.
